# SOARNet: Establishing an older adult focused practice-based research network

**DOI:** 10.1017/cts.2026.10797

**Published:** 2026-07-13

**Authors:** Caitlin Elizabeth Sangdahl, Sara S. Masoud, Vidya Sharma, Meredith Stensland, Cynthia Sierra, Jennifer LaCoss, Angelica Davila

**Affiliations:** 1 Institute for Integration of Medicine and Science, The University of Texas Health Science Center at San Antonio, San Antonio, TX, USA; 2 School of Nursing, The University of Texas Health Science Center at San Antonio, San Antonio, TX, USA; 3 Nutrition and Dietetics, The University of Texas at San Antonio, San Antonio, TX, USA; 4 Psychiatry and Behavioral Sciences, The University of Texas Health Science Center at San Antonio, San Antonio, TX, USA; 5 Geriatrics, Gerontology, and Palliative Medicine, The University of Texas Health Science Center at San Antonio, San Antonio, TX, USA; 6 Family and Community Medicine, The University of Texas Health Science Center at San Antonio, San Antonio, TX, USA

**Keywords:** PBRN, older adult, recruitment, successes, challenges

## Abstract

Older adults experience high rates of chronic disease that reduce quality of life and independence. In South Texas, these challenges are compounded by limited access to culturally aligned care and a high burden of chronic conditions. Addressing these disparities requires infrastructure that bridges research and practice to support the translation of evidence into real-world care. This manuscript describes the establishment of the Supporting Older Adults through Research Network (SOARNet), a population-specific practice-based research network (PBRN) and reports early lessons from its development and recruitment strategies. SOARNet employs a multi-level leadership structure and an Advisory Board to guide research priorities, review member-initiated proposals, and facilitate collaboration among clinical, research, and community stakeholders. Recruitment strategies included informational materials, clinic visits, and targeted email outreach. Since inception, SOARNet has enrolled 61 members representing healthcare clinicians (55.7%), researchers (18.0%), community members (32.8%), and organizations (8.2%), with targeted email outreach proving most effective (*n* = 56). SOARNet has supported a collaborative research study, facilitated member consultations, and contributed to dissemination activities. These findings demonstrate the value of population-specific PBRNs in advancing community-engaged translational research to improve care for older adults. Future priorities focus on expanding partnerships and strengthening culturally responsive, bidirectional research.

## Introduction

The United States continues to face unprecedented growth of the older adult population, including an increase from 58 million older adults in 2022 to 82 million by 2025, marking a 42% increase [1]. Older adults experience higher rates of chronic illness, multimorbidity, and functional decline, among other challenges [2,3]. Worldwide, older adults show consistently high prevalence of disability, frailty, and reduced quality of life related to their health experiences [2,3]. Yet many older adults continue to face limited access to effective, culturally aligned care that may preserve independence and improve health-related quality of life [4,5]. This is particularly true in under-resourced geographic regions like South Texas, where the prevalence of chronic health conditions (e.g., diabetes, dementia, heart disease) remain among the highest nationally and access to timely, culturally responsive care is limited [4–7]. Strained, fragmented, and under resourced health systems leave substantial gaps in care coordination and quality, contributing to poorer health outcomes among older adults including increased hospital admissions, low or no continuity of care, and lower patient satisfaction [8–10].

Addressing these gaps requires infrastructure that bridges research and practice while remaining responsive to community needs. Practice-based research networks (PBRNs) represent a model that embeds rigorous inquiry in real-world settings, producing evidence that is relevant to patients, their families, and clinicians. The PBRN model supports the production of evidence to improve quality of care through engaging the critical perspectives of clinicians and communities in the development of research priorities, studies, and solutions [11,12]. Research that is conducted in close collaboration with direct care clinicians and within real-world settings tends to be more relevant, context-sensitive, and sustainable. These partnerships can lead to interventions that are better tailored to patients’ needs, have greater efficacy, and result in improved health outcomes, particularly for underserved populations where traditional research strategies may fall short [13–15]. We know from past research that older adults are grossly underrepresented in clinical trials, further demonstrating the critical need for practice-based research focused specifically on the older adult population [16–18].

The Supporting Older Adults through Research Network (SOARNet) was established in Fall 2023 at the University of Texas Health Science Center at San Antonio to generate practical and timely evidence to enhance the quality and efficacy of older adult care [19]. At the time of this publication, SOARNet is the only PBRN registered on the Agency for Healthcare Research and Quality database specifically focused on the older-adult population [20].

## Purpose, mission, and member expectations

SOARNet was established to develop and conduct practice-based research aimed at improving the health and quality of care of older adults, with a focus on generating practical, timely, and clinically relevant evidence that can be applied in real-world settings. The membership base reflects SOARNet’s population-specific orientation and includes primary care clinicians, older adults and community members, researchers, and community organizations who are engaged in or committed to improving care for older adults. Researchers in SOARNet represent diverse fields of study, including nursing, medicine, nutrition, social work, public health, psychiatry, and allied health sciences.

Participation in SOARNet is designed to be flexible, allowing members to engage at varying levels depending on their interests and availability. Core expectations include contributing to the development of research ideas, participating in study implementation within practice or community settings when feasible, and supporting dissemination of findings. SOARNet members are able to request free consultations with the Leadership Team and Advisory Board for support on study ideas, implementation, guidance on recruitment, and to seek or propose research collaborations that align with the Network’s mission. All research conducted within SOARNet adheres to established ethical and scientific standards, including Institutional Review Board approval and alignment with Network priorities established by the Advisory Board.

## Methods

### Network structure

**Figure 1.**
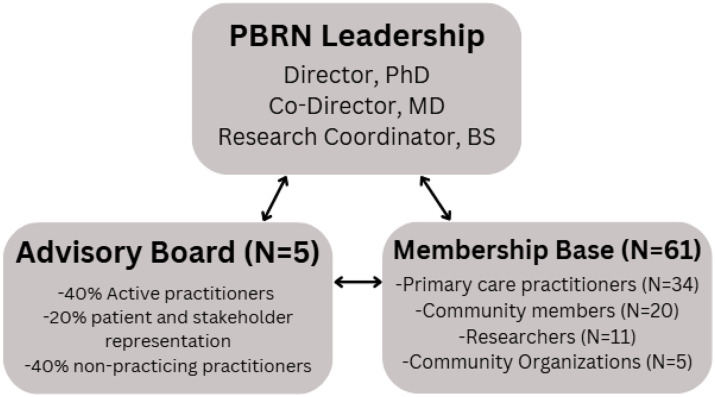
Figure 1 long description. SOARNet structure.

Housed within the University’s Institute for Integration of Medicine and Science, SOARNet is supported through the NIH Clinical Translational Science Award (CTSA) mechanism. Affiliation with the CTSA provided critical infrastructure to support the development of this PBRN, including dedicated effort of a skilled research coordinator at 40% effort. Quarterly PBRN meetings facilitated by the Center Director convened leadership teams of all PBRNs affiliated with the CTSA and promoted collaboration and peer support. The CTSA shared physical infrastructure including office space, meeting rooms, computers, software, and event supplies. Modeled on the structure of other successful PBRNs, SOARNet maintains a tiered, multi-level governance model (Figure 1). Network leadership consists of a research faculty director and a clinician faculty co-director, who jointly oversee Advisory Board activities and guide the growth and productivity of the Network. A research coordinator, provided and funded by the CTSA, works closely with the directors to manage day-to-day operations, facilitate member engagement, and support the implementation of study activities.

The Advisory Board includes five members with varied and overlapping disciplines and experiences: medical doctors (geriatrics, neurology and palliative care), a specialist (dietitian focused on older adult patients), a social worker, family caregivers of older adults, and researchers. They convene quarterly and support the following key functions: monitoring the alignment of Network activities with established priorities, supporting the recruitment and engagement of a diverse membership base, reviewing study proposals submitted by members, and providing strategic and operational guidance for ongoing studies.

### Building the network

We established SOARNet in August 2023, with the first 12 months dedicated to identifying and onboarding a clinical co-director, building capacity of the leadership team, and developing the internal infrastructure required to begin recruitment. The leadership team utilized a REDCap database to support and track member enrollment [21]. With these foundational components of the Network in place, the inaugural advisory board meeting was held in August 2024, during which the enrollment and study proposal forms were finalized and formally approved for implementation. Intentional recruitment efforts began August 2024 upon the first official convening of the SOARNet advisory board. In subsequent advisory board meetings, priority research areas and a guiding protocol for the Network were refined to determine priority areas of focus.

**Figure 2.**
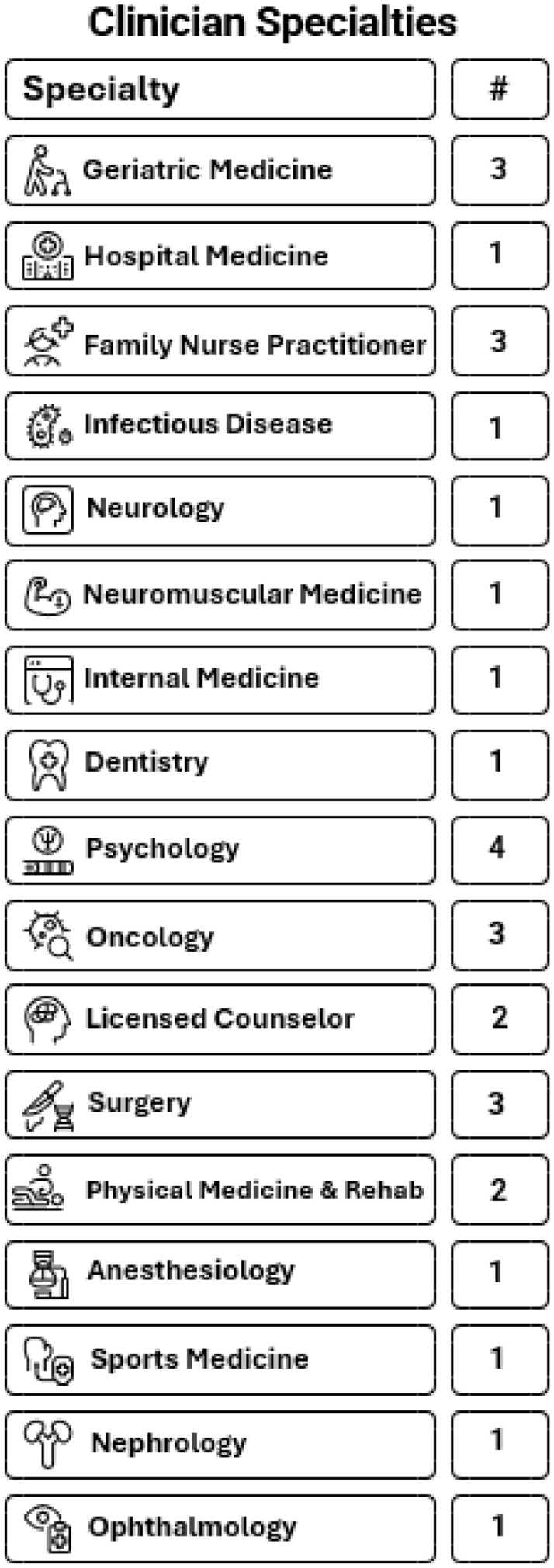
Clinician specialties.

A range of strategies were used to expand the SOARNet membership base, with varying degrees of success. Informational materials, including flyers and brochures, were disseminated to local practices serving older adult patients. To support outreach efforts, the Leadership Team developed an internal database of prospective members and utilized the UT Health San Antonio Find a Provider directory to catalog clinicians by specialty, contact information, and practice location; and a mailing list comprised of community members and researchers from past dementia community engaged research projects the Director had built over time. Recruitment emails were distributed to UT Health San Antonio clinicians, community-based clinics, and organizations focused on older adults and their families. Additional recruitment occurred through word of mouth, dissemination of materials at community events, recommendations from the Advisory Board, and Leadership Team networks. Prospective members completed a 47-item REDCap enrollment form that gathered demographic information, professional affiliation, and level of participation in the Network they would prefer (informational, limited participation, and full participation). Clinicians were requested to estimate the characteristics of their patient populations to inform a general profile of the extended primary stakeholder network represented within SOARNet. Clinician special training, specialty, and membership in other PBRNs were not asked within the enrollment form, but specialty was acquired through the publicly available UT Health Find a Provider database. A wide representation of different specialties is present in the clinician members and allows a holistic view of the older adult population (Figure 2). Except for one clinician, there is no overlap with other PBRNs in the region/state. Due to SOARNet’s unique position within the CTSA, there are many opportunities for cross collaboration among other PBRNs, with a cross-collaborative study currently underway. Additionally, community members were asked if they were a student, older adult, Community Health Worker, or community service clinician or advocate.

## Results

### Recruitment

Results of recruitment efforts conducted between August 2024 and August 2025 are reported (Table 1). SOARNet members (*N* = 61) represent clinicians, community members, community organization representatives, and researchers. Clinicians comprise the majority of the membership (*n* = 34, 55.7%), with nearly one-quarter (*n* = 8, 23.5%) also identifying as researchers. Most clinician members (*n* = 26) practice at UT Health San Antonio, while others are affiliated with the University Health System in San Antonio (*n* = 4), medical practice group (*n* = 3), or the Veterans Affairs Health System (*n* = 2).

**Table 1.** SOARNet member characteristics & demographics Table 1 long description.

Characteristics	*n*	%
**Member characteristics**		
Role* (*N* = 61)		
Clinician	34	55.7
Researcher	11	18.0
Community member	20	32.8
Community organization	5	8.2
**Gender (*N* = 61)**		
Female	47	77.0
Male	14	23.0
**Race (*N* = 57)**		
White	43	75.4
Black or African American	6	10.5
Multi-racial	2	3.5
Other or prefer to self-describe	6	10.5
Ethnicity* (*N* = 48)		
Hispanic	25	52.1
American Indian or Alaska Native	2	4.2
Arab or Arab American	2	4.2
Asian or Asian American	6	12.5
Other or prefer to self-describe	13	27.0
Name of Practice* (*N* = 34)		
UT Health San Antonio	26	76.5
University Health System	4	11.8
Veteran Affairs	2	5.9
Other	3	8.8
Type of community served* (*N* = 29)		
Rural	10	34.5
Urban	26	89.7
Suburban	13	44.8

*Respondents able to select multiple options.

When prompted, clinicians reported they serve predominantly urban-dwelling (*n* = 26, 89.7%) and Hispanic or Latino patient populations (*n* = 29, 93.5%) (Tables 1 and 2).

**Table 2.** SOARNet patient demographics Table 2 long description.

Demographics	*n*	%
**Patient demographics**		
Race* (*N* = 30)		
White	24	80.0
Black or African American	20	66.7
Multi-racial	23	76.7
Other or prefer to self-describe	7	23.3
Ethnicity* (*N* = 31)		
Hispanic	29	93.5
American Indian or Alaska Native	9	29.0
Arab or Arab American	14	45.2
Asian or Asian American	19	61.3
Native Hawaiian or Pacific Islander	10	32.3
Other or prefer to self-describe	6	19.4

*Respondents able to select multiple options.

### SOARNet research

SOARNet serves multiple roles in advancing research within the Network, with a primary focus on providing research support for its members. Through the Network’s infrastructure, members may submit study ideas to the Leadership Team and Advisory Board for review and feedback. Members can also request free consultations with the Leadership Team to explore potential partnerships within or beyond the Network, strategize participant recruitment for PBRN-aligned studies, and address challenges with ongoing projects.

SOARNet additionally facilitates collaborative research. Studies conducted within the Network are supported by the research coordinator in administrative needs, assistance with participant recruitment, management and co-production of study materials, and contributions to writing and dissemination efforts, including presentations and peer-reviewed manuscripts. To date, one collaborative study has been completed that developed and evaluated a nutrition-focused clinician training program intended to improve brain health in older adult Latino patients [22].

### Scientific dissemination

SOARNet has undertaken early dissemination efforts to share network development and research progress. Three posters were presented at local community engagement symposia, two highlighting the establishment of the Network and one describing progress from the collaborative study. In addition, a manuscript reporting results from nutrition–focused study has been completed and published. The training developed has been presented at the UT Health San Antonio family medicine ground rounds with the goal of educating family medicine residents and clinicians. The team has also been invited to present at the 41^st^ Annual Medicine Conference sponsored by the Laredo Area Health Education Center. Together, these activities reflect SOARNet’s commitment to advancing scholarly communication, engaging community and academic audiences, and building visibility for research focused on older adults.

### Challenges encountered during PBRN development:

During the establishment of SOARNet, several challenges were identified related to recruitment, engagement, and resource capacity. Recruitment efforts revealed low overall response rates, particularly among clinicians, despite the use of multiple strategies including targeted email outreach, clinic visits, and dissemination of informational materials. The recruitment strategy that yielded the greatest number of member enrollments was the distribution of targeted, personalized emails sent to prospective members identified through referrals or clinician directories, which yielded 56 new members (91.8% of total recruits). One-time, targeted emails were distributed to 1184 contacts in the prospective directory of clinicians in waves of 100–300, resulting in 27 new members (2.3%). A follow-up distribution of recruitment is pending to those who have yet to enroll. A second directory including community members from the Leadership Team networks (*N* = 818) was contacted using a similar approach, yielding *n* = 22 new members (2.7%). Clinician time constraints also emerged as a significant barrier, with competing clinical responsibilities limiting responsiveness to recruitment efforts and participation in Network activities. Similarly, leadership team capacity constrained the ability to implement more time-intensive strategies such as in-person outreach and sustained follow-up. Additionally, insufficient funding, resource limitations, and uneven representation of clinicians across practice settings and difficulty in engaging new members in the absence of clearly defined action items, emerged as barriers. Methods used to mitigate these challenges are further described in the Discussions section.

## Discussion

This paper described the establishment and early growth of SOARNet, a population-specific PBRN focused on improving research and care for older adults. The Network represents an important contribution to the PBRN landscape, given that PBRNs are most often organized around clinical disciplines or geographic regions rather than a specific population [11,12,20]. By focusing on older adults, an expanding population with complex health needs and persistent inequities in access to culturally responsive care, SOARNet aims to address a critical gap in translational research among this community [2,9].

Recruitment efforts revealed both strengths and challenges. The Advisory Board, composed of individuals with diverse professional and community backgrounds, provided valuable strategic input; however, personalized outreach through Advisory Board members yielded limited success, with only three new members recruited through this approach. Similarly, approaches that have proven effective in other PBRNs including clinic visits and distribution of informational materials did not result in new enrollments for SOARNet [23]. To address this limitation, the SOARNet team prioritized scalable and efficient strategies, particularly personalized email outreach using clinician and community directories. This approach yielded most enrolled members, consistent with other reports suggesting that targeted electronic recruitment is often more efficient than in-person strategies for initiating a network [26] and has been adopted as the primary recruitment strategy moving forward, with plans for repeated outreach waves and expansion of contact networks. However, the overall enrollment rate was modest (<5% of those contacted), reflecting both the challenges of early-stage network development and the time-intensive process of building a membership database. Another potential limitation in recruiting clinicians to SOARNet may be explained by their affiliation with large public sector healthcare organizations. Targeted private practice recruitment strategies yielded minimal results, with only 8.8% of recruited members affiliated with independent medical practices. While independence and autonomy may differ across sectors, a greater representation of clinicians within public sector institutions may indicate more incentive within their roles to be involved in research. Further, having closer proximity to research teams within large healthcare organizations like University Health System or the VA may generate more opportunities for interdisciplinary research collaborations. Limited reach explained by time constraints of the Leadership team and limited resource availability (e.g., printing materials) likely attenuated the effectiveness of this approach. These findings align with prior work demonstrating that recruitment methods successful in one PBRN may not translate directly to another, underscoring the importance of tailoring strategies to local context and population priorities [24,25]. In response to limited resource availability, SOARNet adopted low-burden engagement strategies, including email-based recruitment, flexible participation options, and use of existing professional networks. Additionally, the support of a dedicated research coordinator through the CTSA proved significant in maintaining continuity and managing recruitment and engagement activities.

The Network also encountered difficulty engaging members in the absence of clearly defined roles and opportunities, particularly during its early development phase. Potential members were less likely to commit without a clear understanding of how they could participate or benefit. To address this, SOARNet is working on developing its own By Laws, structured consultation services, and research collaboration opportunities.

These experiences highlight the need for sustained and multifaceted recruitment, as well as infrastructure that supports systematic outreach and follow-up.

## Conclusion/future direction:

Although SOARNet is a new network in a formative stage, its population-specific focus, multi-sector membership, and alignment with the CTSA infrastructure positions it to make a meaningful contribution to older adult research and care. Early lessons demonstrate the importance of tailoring recruitment methods, leveraging institutional resources, and ensuring adequate time and staffing for engagement activities. In addition to increased frequency, diversity, and intensity of recruitment strategies, SOARNet’s current and future priority is to deepen engagement with current members, create opportunities for research partnerships within and beyond the network, and co-develop studies that are responsive to community priorities. Hosting networking events, creating and purchasing branding materials, targeting physician professional societies within Texas, and encouraging current members to spread the word will support the expansion of the Network. Additionally, recruitment can be strengthened by prioritizing clinically relevant research questions and leveraging funding mechanisms, such as Clinical and Translational Science Awards, to enhance member engagement [27]. Incorporating a logic model to systematically map activities and anticipated outcomes aligned with SOARNet’s mission may strengthen overall planning and evaluation of the PBRN [28]. Sustained growth will depend on cultivating bidirectional partnerships between clinicians, researchers, and community organizations, and embedding culturally responsive approaches into all phases of research.

As older adults in South Texas face worsening public health crises, the need for responsive, community-centered research grows increasingly urgent. SOARNet is positioned to enhance clinical and translational sciences through the facilitation of interdisciplinary, patient-centered older adult research. Guided by provider and community member input, SOARNet’s research is rooted in population and place-based priorities and existing scientific evidence. SOARNet seeks not only to generate high-quality, translational evidence but also to advocate for structural change at the local, state, and national levels to support healthy aging. By strengthening community–academic partnerships and fostering inclusive research, SOARNet has the potential to help transform care for older adults while contributing to the evidence base on population-specific PBRNs.

## Figure 1. Long description

The figure shows three rounded grey rectangles in a trianglular layout with arrows going bidirectionally between each rectangle. Within each rectangle, an aspect of SOARNetʼs structure (PBRN Leadership, Advisory Board, and Membership Base) is described in smaller text discussing what or who is included. Figure intended to showcase the structure of SOARNet and the bidirectional interaction between all the 3 participating aspects of the Network.

Navigate back to Figure 1..

## Table 1. Long description

Sixty-one survey respondents were asked to describe their characteristics. Respondents were able to select multiple options. The five characteristics were, role, gender, race, ethnicity, and name of practice. For role and gender, all sixty-one survey respondents responded. Thirty-four respondents identified their role as a provider, eleven as a researcher, twenty as a community member, and five as a community organization. Forty-seven respondents identified as female, and fourteen as male. Only fifty-seven respondents indicated their race, with forty-three identified as White, six as Black or African American, two Multi-racial, and six Other or prefer to self-describe. Of the thirty-four members who indicated their role as a provider, twenty-six indicated the name of their practice was at UT Health San Antonio, four at University Health System, two in Veteran Affairs, and three selected Other.

Navigate back to Table 1..

## Table 2. Long description

A table titled ʼDemographicsʼ with patient demographics categorized by race and ethnicity. The table has two main sections: Race and Ethnicity, each with their respective counts (n) and percentages (%). The Race section includes categories: White, Black or African American, Multi-racial, and Other or prefer to self-describe. The Ethnicity section includes categories: Hispanic, American Indian or Alaska Native, Arab or Arab American, Asian or Asian American, Native Hawaiian or Pacific Islander, and Other or prefer to self-describe. Row-wise data: Row 1: White, 24, 80.0 percent. Row 2: Black or African American, 20, 66.7 percent. Row 3: Multi-racial, 23, 76.7 percent. Row 4: Other or prefer to self-describe, 7, 23.3 percent. Row 5: Hispanic, 29, 93.5 percent. Row 6: American Indian or Alaska Native, 9, 29.0 percent. Row 7: Arab or Arab American, 14, 45.2 percent. Row 8: Asian or Asian American, 19, 61.3 percent. Row 9: Native Hawaiian or Pacific Islander, 10, 32.3 percent. Row 10: Other or prefer to self-describe, 6, 19.4 percent.

Navigate back to Table 2..
